# Veterans’ Perspectives on Fitbit Use in Treatment for Post-Traumatic Stress Disorder: An Interview Study

**DOI:** 10.2196/10415

**Published:** 2018-06-15

**Authors:** Ada Ng, Madhu Reddy, Alyson K Zalta, Stephen M Schueller

**Affiliations:** ^1^ People, Information, and Technology Changing Health Lab Technology and Social Behavior Program Northwestern University Evanston, IL United States; ^2^ People, Information, and Technology Changing Health Lab School of Communication Northwestern University Evanston, IL United States; ^3^ Departments of Psychiatry and Behavioral Sciences Rush University Medical Center Chicago, IL United States; ^4^ Center for Behavioral Intervention Technologies Department of Preventive Medicine Feinberg School of Medicine, Northwestern University Chicago, IL United States

**Keywords:** fitness trackers, patient generated health data, consumer health informatics, stress disorders, post-traumatic, PTSD, mental health, veterans

## Abstract

**Background:**

The increase in availability of patient data through consumer health wearable devices and mobile phone sensors provides opportunities for mental health treatment beyond traditional self-report measurements. Previous studies have suggested that wearables can be effectively used to benefit the physical health of people with mental health issues, but little research has explored the integration of wearable devices into mental health care. As such, early research is still necessary to address factors that might impact integration including patients' motivations to use wearables and their subsequent data.

**Objective:**

The aim of this study was to gain an understanding of patients’ motivations to use or not to use wearables devices during an intensive treatment program for post-traumatic stress disorder (PTSD). During this treatment, they received a complementary Fitbit. We investigated the following research questions: How did the veterans in the intensive treatment program use their Fitbit? What are contributing motivators for the use and nonuse of the Fitbit?

**Methods:**

We conducted semistructured interviews with 13 veterans who completed an intensive treatment program for PTSD. We transcribed and analyzed interviews using thematic analysis.

**Results:**

We identified three major motivations for veterans to use the Fitbit during their time in the program: increase self-awareness, support social interactions, and give back to other veterans. We also identified three major reasons certain features of the Fitbit were not used: lack of clarity around the purpose of the Fitbit, lack of meaning in the Fitbit data, and challenges in the veteran-provider relationship.

**Conclusions:**

To integrate wearable data into mental health treatment programs, it is important to understand the patient’s perspectives and motivations in using wearables. We also discuss how the military culture and PTSD may have contributed to our participants' behaviors and attitudes toward Fitbit usage. We conclude with possible approaches for integrating patient-generated data into mental health treatment settings that may address the challenges we identified.

## Introduction

### Study Motivation

Approximately one in five adults in the United States will experience a mental health disorder each year amounting to 43.4 million adults with a diagnosable mental illness [1]. About 41% of these individuals will receive treatment in a variety of different formats and settings [2]. Across treatment formats and settings when measurement occurs, it typically consists of self-report measures. Traditionally, these measurements use methods such as paper questionnaires and self-tracking journals. However, increasingly, new avenues for collecting data are becoming available through wearable devices such as Fitbits [3] and mobile phone sensors. These devices could contribute to an understanding of a patient’s daily experience and provide some indication of their behavioral and mental health [4,5]. The data from these devices are commonly referred to as patient-generated data (PGD) or patient-generated health data [6,7]. Unlike traditional clinical data, patients, not providers, are responsible for capturing and sharing PGD with health care providers (HCPs). As the penetration of wearable devices and mobile phones increases in the general population, there is an increasing interest in integrating such technologies with mental health treatment [8-10] and leveraging the potential of PGD to transform treatment practices and inform treatment evaluation. However, if such devices and data are going to improve clinical care, it is imperative to understand how they can be integrated with existing care practices to facilitate and enhance treatment.

Mental health issues are particularly prevalent in veteran populations. The rates of post-traumatic stress disorder (PTSD) are much higher for veterans than in the civilian population [11]. Consequently, a variety of approaches are being used to deliver mental health services to veterans [12-15]. One such approach is intensive outpatient programs (IOPs). In these programs, veterans stay at or near the treatment facility for a period of time to receive intensive mental health therapy and, in some cases, wellness interventions. These programs also provide a particularly interesting setting in which to study the integration of PGD into the care process. Specifically, these settings present unique challenges to the integration of PGD compared with other settings such as primary care [16,17] because of the exposure to other patients, frequency of interactions and access to clinicians, as well as environmental differences. Although there is a growing body of research on the use of PGD in primary care settings [18-20], there have been few studies that have examined the challenges of implementing PGD technologies in specialty mental health settings.

In particular, we have little understanding about how wearables and PGD could be integrated into intensive mental health treatment. To address this research gap, we conducted an interview-based study with veterans who had participated in a 3-week IOP. The IOP focused on veterans with PTSD. Participants in this program were given a complementary Fitbit. Because veterans were given minimal instructions on its use within the program, we were able to explore a broad range of motivations without a particular goal of their use in mind. The objective of this study was to understand how patients used their Fitbit and their motivations for either using or not using the Fitbit. This user-focused study is the first step in identifying approaches to meaningfully integrate the Fitbit into mental health treatment.

The paper is organized as follows. In the next section, we present relevant background research related to PGD, veterans, and PTSD. In the Methods section, we present our research questions and describe our methodology. Next, in Results, we identify and describe key motivations that veterans had for using or not using the Fitbits during their IOP stay. We then discuss some key issues we learned about the integration of PGD in mental health treatment in the Discussion section. We conclude with some thoughts on future research directions.

### Background

#### Use of Patient-Generated Data in Mental Health Care

Previous studies on self-tracking in physical health conditions have shown that self-tracking can help patients improve self-management practices [21], motivate behavior change [22] such as increasing physical activity (PA) [23], and identify patterns in health and behavior [24]. Wearable consumer health tracking devices that record PGD are designed with the user as the primary stakeholder; yet increasingly, data from these devices are finding its way into clinical settings in multiple ways [6,7]. For example, such data might enter the clinical setting indirectly through the patient introducing it himself or herself to a provider or directly through application programming interfaces that could integrate the data into traditional clinical practices. Patients may share PGD with their HCPs for a variety of reasons including a desire for a personalized care plan, assistance in making sense of the data, and emotional support. However, there are a number of barriers to fulfilling these expectations [25]. Some of these barriers include a lack of time to review or discuss the data, few HCPs who are qualified to engage with the data, and insufficient mechanisms with which to transfer the data [16].

Although much of the current research on PGD use within the clinical environment has been in the context of physical health treatment such as irritable bowel syndrome [16], diabetes [26], and heart failure [27], there is a growing focus on the role of PGD in mental health treatment. The early work exploring the integration of wearables into mental health treatment largely centered on their use for the adjunctive goal of weight loss [28,29]. The goal of this research was to determine if wearable devices designed to promote PA would be acceptable and feasible in populations in high-risk and challenging populations. As such, these studies focused on individuals with serious mental illness (SMI; ie, bipolar disorder, psychosis, and severe depression). In general, it was found that people with SMI found the devices easy to use and useful for motivation, goal setting, facilitating social connection, and self-monitoring [28]. Indeed, integrating such devices into a lifestyle intervention for individuals with SMI was found to be able to significantly reduce weight below baseline levels [29].

More recent work has focused on using wearables to reinforce mental health treatments. In this work, researchers have focused on the mental health targets that could be reasonably assessed or targeted through such devices. For example, PA has been used in several psychosocial treatments including behavioral activation [30] and exercise indicated for its mental health benefits [31]. A few studies have explored the potential of wearable devices to increase PA as a complement to existing mental health treatments. For instance, one pilot study explored the use of a fitness tracker in the context of group behavioral activation for the treatment of depression [8]. Overall, patients felt positive about the trackers and found that they increased awareness, provided peer motivation, provided opportunities to set and track goals, and provided reinforcement. However, they also noted some challenges, particularly in misrepresentation of information such as stairs climbed. Another pilot study of a lifestyle physical activation intervention for depressed women with alcohol dependence used a fitness tracker to support goal setting and self-monitoring in the intervention [10]. Participants in this study reported high levels of satisfaction with the intervention and with the tracker and reported higher use of PA to cope with cravings or reduce negative emotions. This research suggests that wearables and the data associated with them could potentially benefit mental health treatment.

#### Post-Traumatic Stress Disorder

PTSD is a debilitating condition that can develop after exposure to a traumatic event, that is, an event in which the individual experienced or witnessed actual or threatened, death, serious injury, or sexual violence. The core features of the disorder include re-experiencing symptoms in which the individual has unwanted and intrusive recollections of the event (eg, memories and nightmares), avoidance symptoms in which the individual purposely avoids reminders of the event, cognitive and mood symptoms including persistently low mood and distorted beliefs about the event (eg, inappropriate self-blame), and hyperarousal symptoms in which the individual is on alert (eg, scanning the environment) and on edge (eg, irritability and sleep disruption) [32]. PTSD is also associated with a number of other consequences including withdrawal, isolation, substance use, and suicidality [33,34]. There are several evidence-based treatments that have been shown to be effective for PTSD, including Prolonged Exposure (PE) and Cognitive Processing Therapy (CPT) [35]. These treatments typically involve 10 to 12 weekly 60-min sessions. More recently, evidence suggests that wellness interventions such as mindfulness and yoga may also have positive benefits in the treatment of PTSD [36,37].

Rates of PTSD in military personnel are higher than in civilian samples, with a recent meta-analysis indicating that 23% of veterans returning from Operation Enduring Freedom and Operation Iraqi Freedom suffer from PTSD [38]. However, uptake of evidence-based treatment among veterans and military service members is poor. For example, one study found that only 4% to 14% of veterans received any sessions of an evidence-based psychotherapy during the first 6 months of treatment at a Veterans Affairs specialty PTSD clinic [39]. Moreover, among those that do initiate treatment, evidence suggests that almost 40% terminate before receiving therapeutic benefit [40]. Poor accessibility and avoidance appear to be key barriers to PTSD treatment for veterans [41-43], indicating that novel treatment approaches are needed that can overcome these issues. An increasingly popular approach is to deliver evidence-based PTSD treatment intensively, that is, daily treatment over several weeks with patients living at or near the treatment site, with the goal of reducing external distractions and practical barriers to treatment and providing less opportunity for avoidance [44]. These treatments also allow for the integration of multiple treatment modalities, including wellness interventions, to support treatment adherence and enhance treatment outcomes.

#### Potential of Patient-Generated Data in Post-Traumatic Stress Disorder Treatment

There are several reasons that the integration of wearable devices into the treatment process might help to promote the successful treatment of PTSD. First, a meta-analysis evaluating four randomized controlled trials (N=200) showed that PA was significantly more effective than control conditions in reducing PTSD symptoms [45]. A recent study by Powers and colleagues [46] showed that the addition of exercise to PE significantly augmented treatment outcomes relative to PE alone. The exercise augmentation group also showed enhanced brain-derived neurotrophic factor relative to the PE alone group, suggesting that the benefits of exercise may be because of an enhanced capacity for learning and memory.

Second, in addition to the capacity to support PA, other functions of wearable devices might also help to support treatment success. Sleep disruption is one of the most common and distressing symptoms of PTSD [47,48]. Yet, evidence suggests that front-line treatments for PTSD do a poor job of improving sleep [49-51]. Largely, clinicians rely on self-reported sleep disruption, which is known to be flawed and is often mistrusted by providers [52]. Thus, better understanding of a patient’s sleep patterns and sleep quality using wearable device data could help to inform clinical decision making in the context of PTSD treatment, including the use of adjunctive sleep interventions.

Finally, beyond the specific functionality of wearable devices, it is also possible that the integration of PGD into the treatment setting could help to build rapport between the patient and provider [25]. Therapeutic alliance has been shown to be an important predictor of treatment outcome for PTSD [53].

#### Summary

As highlighted in this section, there is growing interest in the role of wearables and PGD in mental health treatment. In particular, although the use of PGD in PTSD treatment holds promise, more research is needed to investigate how PGD can be integrated into an intensive outpatient care setting for the treatment of PTSD. We were able to take advantage of the opportunity to address these two questions through interviews with veterans who attended a 3-week IOP for PTSD in which Fitbit devices were routinely deployed. Consequently, for this study, we had the following two research questions:

Research question 1: How did the veterans in the intensive treatment program use their Fitbit?

Research question 2: What are contributing motivators for the use and nonuse of the Fitbit?

Driven by the sociotechnical perspective [54] in which the meaningful use of a technological system is highly dependent on its interactions with people and processes, our study explores how the device was used in the program and how veterans perceived the opportunities and barriers to its potential usefulness and integration.

## Methods

### Setting

Participants were recruited from a database of veterans who had completed the IOP for PTSD at the Road Home Program located at Rush University Medical Center. The IOP consisted of a 3-week, daily treatment program that provided evidence-based treatment for PTSD. Each cohort of patients consists of 10 to 13 veterans.

Treatment at the IOP was multifaceted: the core elements of the program included daily individual and group CPT [55], as well as group mindfulness and yoga. Veterans also received a number of secondary intervention components including fitness, nutrition, psychoeducation on relevant topics, medication management (as needed), case management, art therapy, and acupuncture (optional). For assessment purposes, veterans were asked to complete four clinical survey assessments regularly to track their symptoms of PTSD (PTSD Checklist for Diagnostic and Statistical Manual of Mental Disorders, Fifth Edition, PCL-5) [56,57], depression (Patient Health Questionnaire-9) [58], negative cognitions (Post-traumatic Cognitions Inventory) [59], and guilt (Trauma-Related Guilt Inventory) [60].

As part of the treatment program, veterans received a complimentary Fitbit Charge HR during their first day of treatment. The Fitbit Charge HR has passive activity tracking and sleep tracking capabilities including step counting, heart rate monitoring, and sleep monitoring. The companion Fitbit mobile app allows for manual input of weight, food, and water intake, as well as the review of all logged data. The use of the Fitbit was completely voluntary.

In a 15-min session, research assistants distributed Fitbits to the veterans and assisted with device set-up including syncing with a mobile phone if the veteran had one. Veterans were informed that the Fitbits were being provided because the IOP was interested in learning how health and activity levels changed over the course of treatment and after patients returned home. Veterans were also given a brief tutorial regarding the functionality of the Fitbit and were told that they can raise any questions about their Fitbit in their nutrition class the following morning. Veterans were also given the option of sharing their data for future quality improvement initiatives aimed at determining the value of the Fitbit data. If they agreed, they provided their Fitbit account log-in information to Road Home Program staff, who downloaded the information from their account at the end of the 3-week stay. In total, they were able to download Fitbit data for 73.8% (93/126) of patients who attended the IOP and received Fitbits.

### Recruitment and Participants

The program staff emailed recruitment messages to veterans that completed the IOP within 12 months before the study and had agreed to have the program store their Fitbit data. The first author also conducted in-person recruiting at the IOP for the ongoing cohort at the time. We interviewed a total of 13 participants (Table 1). Due to the in-person recruiting efforts, 5 of 13 (38%) of our participants came from the ongoing cohort. The longest a participant had been out of the IOP was 9 months. Our participant sample represents approximately 13% (13/96) of all veterans that completed the IOP in the 9 months before the interviews.

The participants consented once online and also over the phone before the interview. They did not receive compensation. This study received institutional review board approval from the authors’ home institutions.

An overwhelming majority of the study participants were men (11/13, 85%). Their ages ranged from 30 to 61 years with a mean of 41 years (SD 10). The majority of study participants (9/13, 69%) entered the program with a PCL-5 score that classified their PTSD symptoms as “severe,” and the remaining participants had a symptom classification of “moderate.”

### Data Collection

In August and September 2017, we conducted 13 semistructured interviews with veterans and service members (hereafter referred to as “veterans”) who had previously completed the IOP at the Road Home Program. Each interview lasted approximately 60 min. We employed a semistructured interview guide to allow for consistency across participants yet flexibility to further explore topics as they arose during the interviews. The phone interviews were conducted to ease travel burden on participants and allow for recruitment of veterans who did not live in the local area. All interviews were audio-recorded and transcribed by a member of the research team or by a company independent of the research team. Coding was conducted based on these transcripts. Data collection continued until data saturation was reached and interviews no longer revealed new or surprising information [61,62].

The interview guide was organized around our two research questions. For our first research question (research question 1: How did the veterans in the intensive treatment program use their Fitbit?), we asked participants about their experiences with self-tracking, both Fitbit and non-Fitbit, before and during their time in the program. For our second research question (research question 2: What are contributing motivators for the use and nonuse of the Fitbit?), we asked participants about their motivations and desired outcomes from using the Fitbit. For participants that used the Fitbit for self-tracking, we also asked what they did with their self-tracked data, including their data sharing practices and the actual outcome of tracking. Finally, we asked the participants about their attitudes toward self-tracking and additional envisioned uses of the Fitbit within the IOP. The full script for the semistructured interview guide is available in Multimedia Appendix 1.

**Table 1:** Participant demographics.

ID	Age, years	Sex	Race	Number of months between IOP^a^ completion and interview	Branch	PTSD^b^ severity at baseline^c^
P1	41	Male	African American	3	Army	Severe
P2	57	Female	White	2	Army	Moderate
P3	52	Male	African American	4	Marine	Severe
P4	34	Male	White	6	Army	Severe
P5	41	Male	Other	4	Navy	Severe
P6	61	Female	African American	4	Army	Moderate
P7	38	Male	White	6	Army	Severe
P8	37	Male	White	9	Army	Moderate
P9	30	Male	White	0	Army	Severe
P10	36	Male	African American	0	Marine	Severe
P11	34	Male	White	0	Army	Severe
P12	34	Male	White	0	Army	Moderate
P13	42	Male	White	0	Army	Severe

^a^IOP: intensive outpatient program.

^b^PTSD: post-traumatic stress disorder.

^c^PTSD severity at baseline based on PTSD Checklist for Diagnostic and Statistical Manual of Mental Disorders, Fifth Edition (PCL-5) scores. Scores of 37 to 49 correspond to moderate and 50 to 80 correspond to severe.

**Figure 1:**
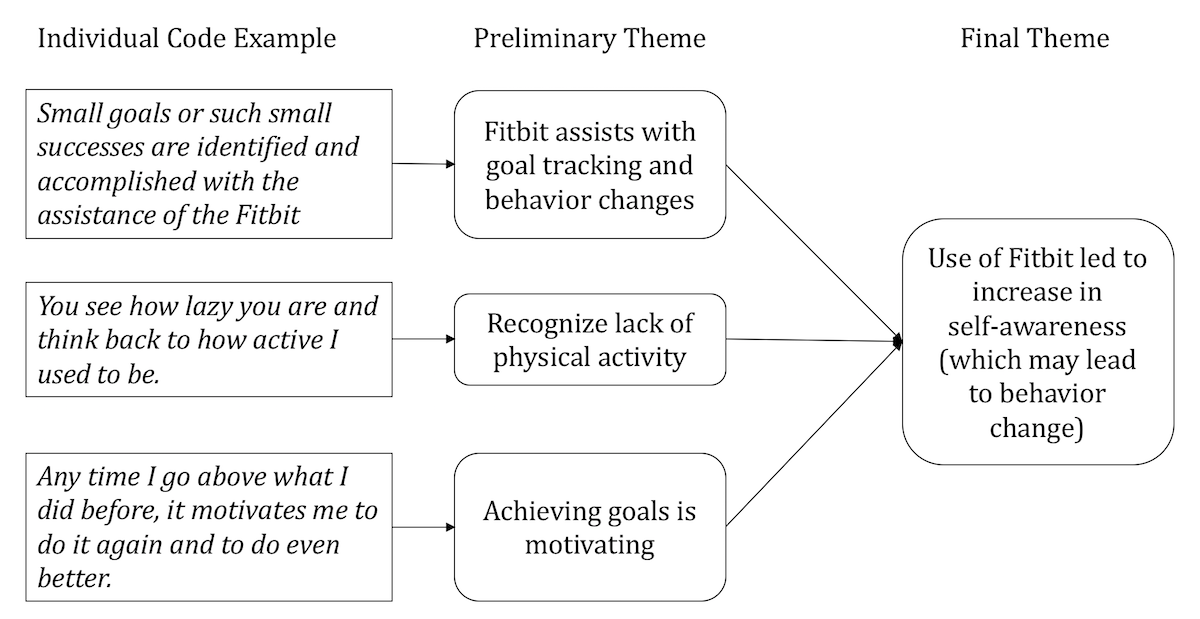
Example interview coding process.

### Data Analysis

The transcribed interviews resulted in 360 pages of electronic transcription data. For our data analysis, we used a thematic analysis approach [63]. This approach involves becoming familiar with the data, systematically identifying individual codes, and organizing these codes into broader themes (Figure 1). We used a single coder approach in which the first author iteratively identified individual codes during and after data collection, refining themes throughout the study. Single coder approaches are methodologically sound when they include checks on validity and reliability. For our purposes, validity and reliability were promoted using a peer-checking process in which the first author iteratively reviewed a selection of code definitions and raw text with the second and final authors. This process is commonly used in qualitative research [64,65].

To outline our approach more specifically, the first author began analysis by systematically reviewing each transcript multiple times. Individual codes were created each time a participant mentioned use or nonuse of the Fitbit (eg, participant says he identifies small goals and the Fitbit helps him reach them). Codes were also created when participants mentioned contexts surrounding use of the Fitbit (not pictured in Figure 1; eg, a veteran talking to the director of the program about Fitbit data). Individual codes were then grouped into preliminary themes based on similarities (eg, Fitbit assists with goal tracking and behavior changes). Memos were used to keep track of emerging common themes or developing codes. Finally, the preliminary themes were grouped into broader final themes (eg, use of Fitbit led to increase in self-awareness (which may lead to behavior change). The themes identified were grounded in the data itself as opposed to existing theories as per the thematic analysis approach. This approach allowed us to openly explore the individual contexts and motivations through which participants chose to engage or disengage with their Fitbit.

## Results

### Motivations for Use

In this section, we describe three major motivations for veterans to voluntarily use the Fitbit during the IOP: increased self-awareness, its contribution to supporting social interactions, and a desire to give back. In these findings, a recurring influencer of these motivations was the intensive treatment context in which patients were provided and used the Fitbits. Relatedly, veterans’ motivations to use the Fitbit emerged only after receiving the complementary device and may differ from motivations of users who purposefully purchased a device on their own.

#### Increase Self-Awareness

The Fitbit’s ability to track a variety of physical data increased the self-awareness of some of the veterans about their activities. In turn, this increased self-awareness provided veterans a mechanism to better understand their unhealthy behaviors. For instance, one veteran noted that the Fitbit helped him better understand his sleep patterns. He stated the following:

[The Fitbit] really showed me how much sleep I was getting because I really didn’t think it was that bad until I got the Fitbit.

P3

The Fitbit data provided the veteran with “concrete” evidence of previously unknown problems.

Another effect of increased self-awareness is that participants started to formulate goals and change their behaviors to meet those goals. One veteran (P3) stated the following:

...When I got the Fitbit and I started putting 10,000 steps down as my goal, the first couple of times I didn’t get it and I said “Well maybe I should change” or I said “No maybe I need to step up to the challenge” so that was how it started.

P3

For this veteran, becoming aware of his current state of physical fitness when he failed to reach his step count goals led him to adopt new habits such as walking between class and the dormitory and taking the stairs instead of the elevator.

Although increased self-awareness led mostly to positive behavior change, it is important to note that there were instances where this was not the case. For instance, one veteran (P12) reported that a rising heart rate often accompanied anxiety that occurred while being in a crowd. So, he would review his heart rate data and attempt to recall the context around when his heart rate is elevated. He stated the following:

Basically yeah just try to figure out “Hey the pulse was getting high—was I doing this or going there?” It might be because of anxiety or whatnot so I would just try and figure things out before I go back to that place again or whatnot.

P12

Thus, although the Fitbit aided him in being able to associate changes in heart rate with potential cues such as specific activities or his surroundings, P12 used this increased awareness to avoid situations that led to an increase in his anxiety. Avoidance is viewed as counterproductive to treating anxiety [66]. So, in this case, the increased self-awareness actually led to a negative behavior change from a treatment perspective. However, this association could be used in P12’s therapy to help address the why his anxiety increased near those settings.

#### Supporting Social Interactions

During their 3-week stay, veterans interacted with their peers and clinicians daily. Consequently, one of their motivations for using the Fitbit was that it supported a variety of social interactions including competition, support, and conversation.

One of the many ways that veterans interacted with each other was through competition. Many of the participants stated that as veterans, they were trained to be competitive, and that is how they often interacted with each other in the military. During the IOP, this often took the form of a step-count competition, as illustrated in the following quote:

People in the military always like to make everything into a little competition so it was just kind of one of those, you know, over the weekend or something like that, it’d be like “Oh well how much have you been walking?” and kind of pick on each other and just kind of using it as a I don’t even know what the word is...kind of give us something to joke around about, you know. To kind of help break the mood up every once in a while you know, or kind of just take our attention off of what was going on.

P4

Increasing daily PA was a shared goal among many veterans. Therefore, they created online communities on the Fitbit platform for their cohort where the veterans could participate in step count challenges. These communities had leaderboards to highlight who took the highest number of steps in the day or week.

Second, the Fitbit also promoted supportive interactions between veterans and between veterans and their clinicians. For example, other veterans supported P2 who had a personal goal of taking one more step than she took the previous day in an effort to increase her PA, as illustrated in the following quote:

What I try to do is I try to do one more step the next day but then sometimes I get depressed and I’m like “Oh I’m fat, what difference does it make?” but I have a couple of the ladies from the program and you know they’ll say “Okay we’re going to do this together this week” and then I’m like “Oh okay” and then I’ll try to get back into it.

P2

In this example, she tracked her steps using the step count feature on the Fitbit and shared the results with her peers. They used this data to encourage her in her progress toward her goal.

Although the Fitbit and its data were not formally integrated into the IOP program, veterans did use their Fitbit data in their interactions with clinicians. For instance, a participant was able to retrieve the number of hours he actually slept instead of just relying on his recall when his clinician asked him about his sleep, as illustrated in the following quote:

I have the data. It says I haven’t slept a lot versus I slept 4 hours and 28 minutes on average this week. In the medical field people are like hey 7-8 or 6-8 hours is the normal. You know, to be more specific. I bet if I went through my doctor or something like that they can be like ‘Huh we need to look into it’ hopefully.

P10

The Fitbit could allow P10 to more accurately provide specific data to the clinician. This could make his conversation with the clinician more meaningful to him.

Once the Fitbit made the veterans more aware of their issues, some of them would initiate dialogue with their therapy team about these problems. In one example, a participant who had been wearing his Fitbit described how he started a conversation about emotion management by reviewing his heart rate data with the nurse on staff and his clinician. He was ultimately able to tackle some issues through the mindfulness training he received, and this reinforced the usefulness of the Fitbit for him. He stated the following:

...so that’s how it all came full circle and why I continue to wear that app, or this Fitbit is...these are the things that it’s just multifaceted and if we didn’t have all those different programs all together at the Road Home, then I don’t think the lightbulb would have clicked.

P7

For P7, discussing his Fitbit data with the different clinicians gave him a better understanding of how his physical health impacted his mental health. The notion of physical and mental connectedness is further examined in the Discussion section.

#### Giving Back

Several participants stated that they hoped their Fitbit data would benefit future veterans, move research forward in the treatment of PTSD, and support the development of the IOP. One participant described his contribution of data in the following way:

By sharing the data, it makes me feel like I’m helping contribute to other people’s health. By helping to get them better by allowing then to look back and see what worked and didn’t work and how well it’s affected me.

P13

Other participants stated that they were particularly interested in research that could improve PTSD interventions for veterans, as illustrated in the following quote:

...I hope that using our information that they’re keeping track of helps the program down the road and help really kind of link some of the things between PTSD and physical wellbeing and things like that to kind of help guide physicians and dieticians and things like that to kind of what would be better for the veterans instead of kind of going off of I guess what I would call the standard type thing of “Oh hey you need to cut back on red meats. You need to start eating more vegetables.”

P4

This example also highlights the potential relationship between physical and mental health that participants frequently mentioned during the interviews.

Participants also reported using the Fitbit to express gratitude for having the opportunity to participate in the program. One participant emailed a screen capture showing the number of steps he took that day to the director of the program. He stated the following:

I wanted them to know that I was taking the program seriously. Like what you gave me—the resources you gave me—I used them. That’s big with me...I wanted them to know that physically, I’m doing this thing as well as mentally.

P3

P3 and a few other participants believed that taking the program “seriously” (fully engaging) meant not only putting in effort to pay attention in therapy and do homework but also wearing the Fitbit.

### Motivations for Nonuse

In the previous section, we described motivations for using the Fitbit. However, not all participants used the Fitbit. In this section, we identify three major reasons that the veterans did not use the Fitbit or certain features of the Fitbit.

#### Lack of Clarity About Purpose

Participants who did not use their Fitbits questioned the benefits of tracking for their self-improvement or as part of their therapy. Because there was little direction from the IOP about the role of the Fitbit in the program, many veterans did not understand its purpose. One participant who described himself to be in good physical shape abandoned use of his Fitbit after the first week at the program. He stated the following:

I was the only active duty person in the class and some of them were...some of my peers were overweight and some of them had injuries so for me, at my age, I go off of our physical fitness test and our height and weight standards and that’s how I gauge myself so I mean I can’t compare myself to somebody that’s not either required to exercise or has an injury.

P5

P5 primarily thought of the Fitbit as providing him information about his physical status. It was not made clear to him how the Fitbit could support the broader goals of the program.

The IOP’s approach to treatment was holistic and incorporated trauma-focused therapy with physical wellness activities, but many veterans did not see this link, which affected their use of the Fitbit. When one participant (P13) was asked why he did not discuss his Fitbit data with his clinician, he explained that his priority was therapy. Therefore, he wanted to focus on the homework assigned by his individual therapist that he believed would more directly address his mental health concerns. He stated the following:

Yeah the therapy is what we were there for versus the physical. I mean, this is for PTSD and I know my physical injuries were a lot but we were talking more about mental so that was what I was focused on. I think the little bit of physical stuff we got is a big help but I don’t think that being in that program we were worrying about the physical aspect. I know they were trying to teach us stuff and that was good but we were so wrapped up on the mental that the little bit of physical stuff we were doing was second and I think that’s where we needed to be.

P13

The belief that physical and mental wellness were connected varied between participants. Some participants saw them as disparate components of health, but others believed they were intertwined. P7 describes how providers at the IOP taught him how physical and mental health were connected, as illustrated in the following quote:

...I was always just thought like okay physical activity means physical health but there they spent the time to explain that physical activity does mean physical health but it could also mean mental health for a lot of different reasons and so that really struck a chord with me.

P7

Not all participants left the program with the same belief as P7, and the extent to which veterans believed that physical fitness influenced their mental wellness was a motivator to engage with the Fitbit or a reason to disengage.

#### Lack of Meaningful Data

The veterans were not given any formal training on how to use the Fitbit or interpret the data, and this inability to derive meaning from the data discouraged some participants from using the Fitbit. For instance, P1 was unable to decipher how the Fitbit tracked his sleep and did not understand the data as it was presented in the mobile app. Consequently, he abandoned the sleep tracking feature:

Tell me a little bit more about sleep because it’s interesting that you kept a sleep journal for a little bit but you didn’t track sleep with your Fitbit from what I understand

Interviewer

No I did not. I tried to a couple of times. Honestly maybe because I didn’t understand it. How can it tell when I wake up or if I was asleep or?..I didn’t get any detail out of it...I didn’t look at it in detail to see how it really tracks my sleep like how accurate is it? So I chose not to.

P1

As the example highlights, Fitbits may reduce the burden from manual tracking, but if veterans do not understand or trust the data, it could be difficult for them to be motivated to use it.

However, even when participants know how to use it, the Fitbit may not capture the issues that they want to better understand. In the following example, P4 discusses how the Fitbit’s measures for sleep does not reflect the fact that he experiences night terrors, which is common in patients with PTSD [67].

I mean they were useful but not at kind of what I was hoping for as far as trying to show how restless I was, you know, or my...hope...trying to think how...like kind of help try to track my heart rate or anything like that because it was tracking just movement during sleep and not really heart rate or anything like that.

P4

So it wasn’t quite useful for sleep because it was only tracking movement and not heart rate so you’re saying if it did track heart rate during sleep that would have been more telling?

Interviewer

I would think so, you know or some way to kind of show, you know something other than just movement because I have, with my night terrors, I don’t know what it’s called right now pretty much I would be sweating really bad even though...like I’d be wrapped up in a blanket feeling cold but sweating at the same time.

P4

The Fitbit did not have the mechanisms to detect night terrors, and therefore, tracking sleep using the Fitbit was not perceived to be valuable. For these participants, tracking a particular behavior could have proved to be meaningful to them; however, the Fitbit itself was unable to meet the user’s aims.

#### Challenges in the Veteran-Provider Relationship

The rapport between a therapist and patient is critical for effective therapy [53], yet several participants mentioned that veterans have an inherent distrust of HCPs, which made it difficult for them to share information including Fitbit data. P8 explains how veterans may be hesitant to disclose information to clinicians to avoid feeling “judged” by someone who has not experienced war, as illustrated in the following quote:

I really upset some therapists when I told them I could get more out of a soldier at a smoke pit saying you can get from an office...They [therapists] weren’t there. They didn’t experience any of it. They may have secondary PTSD from hearing everybody else’s stories but it’s that first-person understanding that helps.

P8

When asked for his thoughts on veterans initiating a conversation regarding the Fitbit data with clinicians at the IOP, he viewed the relationship in a more adversarial way, which prevented him from wanting to share information. He stated the following:

“They’re not telling us what’s happening. How can we help these guys if they don’t open up to us?” It’s a battle that a lot of these therapists are having with us. I don’t really understand how we could initiate something like that because I’ve been actively trying to combat PTSD for the last nine years in October.

P8

Other participants with a similar hesitation suggested that it’s best if clinicians initiate a conversation regarding the Fitbit data. A few mentioned that they would openly share their data if they were directly asked, as illustrated in the following quote:

Who would I share it with? Nobody ever asked. If they had asked I would have shared it. But they didn’t ask. I mean even when they ask for the blood tests and stuff, they had some people that had a hard time taking blood but even then they said “No no, keep trying”...and I don’t know what information they need or who it is. I would have shared it with them.

P2

The motivations surrounding how a veteran could use the Fitbit data in their sessions with clinicians are complicated. However, before this data can even be used, there has to be a level of trust built between the veteran and therapist.

## Discussion

### Principal Findings

This study contributes to a growing body of research on PGD use in mental health care. Although our investigation took place in a single setting in which Fitbit Charge HR devices were distributed, many of our findings were consistent with findings from studies exploring people engaging in self-tracking in other settings, as well as tracking enthusiasts often referred to as “quantified-selfers.” For example, self-awareness is a common motivation that is not unique to our veteran population [68,69]. Previous studies on individuals that track health information, financial information, or other life information found that tracking plays an important role in how people learn about themselves and use that information to change their behavior [24,68]. Similarly, we found that veterans who were motivated to use the Fitbit often used to it learn about their current health status, and in some cases this led them to change their behaviors. However, because this was the first study to explore motivations of individuals undergoing mental health treatment, we also identified some unique issues in this study. For instance, in previous studies of PGD, patients were often eager to share their data with clinicians [25]. However, in this study, some veterans had difficulty sharing data with their clinicians because of their concerns that the clinicians could not understand them because of the clinician’s lack of experience in the military. This hesitancy resulting from different backgrounds was not found in other studies. Furthermore, although this research reaffirms findings of PGD research in other settings [16,17], it also expands it into a domain that has not been studied before—veterans who engaged in an intensive mental health treatment to help them manage their PTSD.

In the rest of the discussion, we turn our attention to the military culture, PTSD and Fitbit use, the integration of PGD into mental health treatment settings, and the transformative opportunities that are possible with PGD.

### Military Culture, Post-Traumatic Stress Disorder, and Fitbit Use

Veterans have a shared experience of military life and culture that differentiates them from the general population [70]. Military life is more interdependent than civilian life, with military personnel undergoing training that emphasizes a commitment to each other and service to their country. It also has ranks and promotions that makes a social hierarchy clearly visible. Participants commonly identified features of the Fitbit that could be tied to military values or could leverage characteristics of “military people.” For example, many participants noted that challenges such as the step competition appealed to the competitive nature that is common among individuals who had served in the military. Furthermore, the close camaraderie that is part of the military is reflected in the participants’ willingness to use the Fitbit to benefit future veterans of the program. These differences between military and civilian life have led to calls to tailor health care specifically to this population in what is deemed veteran-centric care [70]. This model mostly focuses on acknowledging issues that might affect behavior such as complex deployment and reintegration needs or understanding of common issues facing veterans including PTSD, traumatic brain injuries, depression and suicide, or amputations and rehabilitation care. Fitbits could play an important role in this model because of the physiological data it could provide clinicians about the veterans that could be used to better understand their problems. Our findings highlight some of the motivations that are drawn from the military culture that affected how veterans were motivated in using the Fitbit.

The interdependent nature of military culture might have been accentuated by aspects of the IOP program. Participants in this program come from around the country to spend 3 weeks receiving treatment for PTSD, leaving their lives, and sometimes their families behind. Although this experience may be familiar to military personnel, it is still different from many other health care treatment settings that treat mental health issues such as PTSD. Many IOP participants described a sense of camaraderie with peers from their cohort. This camaraderie could be leveraged to encourage them to use the Fitbit and be attentive to the data. Furthermore, this camaraderie could also help increase veterans’ awareness of each other that could be used for behavior change in the same way that participants described self-awareness for behavior change. For example, if veterans became more attuned to how others were sleeping, it might increase empathy for peers when going through a day’s treatment after a poor night’s sleep or make the individuals more attuned to cues linked with poor sleep (eg, emotion regulation, attention, and focus). Facilitating awareness of others might contribute to a culture of discussion around links between insights gleaned from one’s data and emotional and physical health, which could in turn also facilitate sharing and use in their treatment.

Our findings indicate some promising future considerations for PGD and its contribution to helping address mental health issues such as PTSD. First, veterans were motivated to use the Fitbit to increase their self-awareness that could ultimately lead to behavior change. Self-monitoring is a common treatment element used in a variety of effective mental health treatments with the goal of promoting self-awareness [71]. PGD could support current practices of self-monitoring or even open up new opportunities for self-awareness. Second, PGD might be able to better quantify aspects of mental health conditions by providing a more “objective” history of this information than patients could provide. As participants indicated, they could discuss physiological avoidance with the example of heart rate data or could potentially more accurately report their sleep quality or quantity on a given night. Finally, mental health treatments might have benefits that extend beyond mental health symptoms alone. In efforts to reduce assessment burden, clinicians and researchers are often limited as to the number and breadth of questions they can ask patients. PGD could help clinicians better understand the broader impact of treatment and help clinicians better tie treatment activities to meaningful changes in a patient’s life.

### Integrating Patient-Generated Data Into Mental Health Treatment

There are a number challenges that we must address as we consider the best approaches for integrating PGD into care settings for mental health treatment. We have to consider issues related to processes, policies, and technologies. Furthermore, we need to better understand the issues related to the growing focus on combining physical and mental health treatments [72,73].

#### Processes, Policies, and Technologies

Researchers have identified a variety of organizational [74] and individual factors [75] that affect the implementation and use of new technologies. These issues include designing policies and processes to support the use of the technology along with developing training and implementation strategies for the technology [76].

First, before any technology such as Fitbits are implemented in a care setting, there has to be clear policies in place to handle issues such as privacy and sharing of data. This is particularly important when dealing with wearable devices that continually collect data about the individual [77]. The concerns about privacy were raised by the veterans, who were unsure about whether clinicians were allowed to look at or discuss the Fitbit data because of privacy rules. Therefore, in a setting such as the IOP, it is important to develop clear policies about privacy-related issues such as who can see the PGD, how can patients change their privacy preferences, and who owns the data.

Second, it is important to ensure that the technology is integrated into the workflow of the organization. Researchers have identified a variety of challenges that arise when technologies are not properly integrated. These challenges include resistance to adoption, development of workarounds, and lack of use [78]. One of the primary challenges to effectively utilizing the Fitbit was the lack of any process to integrating it into the IOP workflow. Besides a short training session on how to use the Fitbit, the veterans were not provided with any details about how the Fitbits could be used in their care process during their 3-week stay. Furthermore, there was no formal process to share the data with the clinicians. The lack of processes for incorporating the Fitbits into the treatment plan for the veterans was a major barrier for use. As our findings noted, one major disincentive was the lack of a clear purpose. This lack of purpose led to the lack of clear processes in the IOP.

Finally, the successful implementation of any technology requires a well-designed implementation and user training plan [76]. When this does not happen, technologies often fail, or the adoption of these technologies are much slower than what was anticipated [79]. In the IOP, the veterans were provided with minimal training on how to use the Fitbit. Furthermore, the lack of an implementation plan for incorporating the Fitbit into the IOP led to confusion about its benefits. As one veteran noted, he did not see the need to use the Fitbit because he was not dealing with physical issues but rather mental ones. Because of a lack of training, it was not clear to the veteran how the Fitbit fit into the broader treatment plan. The lack of a clear implementation plan and effective user training increased the barriers to using the technology.

#### Building Connections Between Physical and Mental Health

The use of self-tracking devices for mental health represents both a growing interest in technology for mental health [80] and trends in health care generally to combine physical and mental health treatment in integrated behavioral health models [81]. Indeed, our findings suggest that a key aspect of facilitating this connection is to increase patients' awareness of how using these devices to track physical data can actually support mental health care. Participants who did not see this connection were unmotivated to use the Fitbits because of their views that they were in the program to work on their PTSD as opposed to physical health concerns. This could have been mitigated through more direct instructions as to how to use the Fitbit and data gained from it in this clinical context. In some cases, although there were no clear instructions, participants spontaneously made these connections, finding relationships between things such as heart rate and anxiety, sleep and type of therapy received (eg, sleep better on days with art therapy), and PA and mood. However, many of the veterans did not see the connection, which led to underutilization of the Fitbit. Consequently, organizations who want to utilize self-tracking tools such as the Fitbit must more clearly connect the relationship between physical and mental health.

### Transformative Opportunities With Patient-Generated Data

PGD has the potential to create new opportunities and new conversations in mental health treatment. Our theme of veteran-provider relationship is an important finding because the relationship between patient and provider is a critical determinant of successful mental health treatment [82] and has been found to predict improvement in exposure-based treatments for PTSD [83,84]. Along these lines, PGD could benefit this relationship by providing concrete examples that providers could use to tailor their treatments and thus, allow providers to more effectively treat veterans. Additionally, sharing PGD could start conversations that build trust and understanding between patient and provider by allowing the provider an additional window into a patient’s life. However, the use of such data and devices in clinical practice and clinical research does raise a host of ethical questions [85]. Yet, dealing with these questions is likely a necessity given the increased attempts toward using this data in the health space.

PGD also represents a different type of information entering the clinical context for mental health treatment. As mentioned earlier, mental health assessment is largely based on self-report, reflective measurements, and questions. Many providers open their sessions by asking patients about what has happened since the last time they met. PGD might facilitate a different type of interaction—one that explores patterns or specific values in data collected outside the session and in real-world contexts. In light of this, it would be useful to think about how PGD can expand or change thinking toward clinical data rather than viewing it as an unobtrusive and passive method for replacing that data. As such, future work should start from the viewpoint of what PGD can offer by itself, and interview studies, such as this one, are valuable tools to guide such work.

### Limitations and Future Work

Given the dearth of research in this area, this study was an important step forward. However, as an early step into a new research area, the study had several limitations that could be addressed in future work. Our sample was self-selected in several ways, including only using veterans who agreed to have Road Home download and store their Fitbit data. As such, our results might be oriented toward those who might be more interested in using the Fitbit rather than the average veteran. However, we do note that overall 73.8% (93/126) of those receiving Fitbits opted to provide their data, so this still represents a large proportion of veterans treated in this program. Furthermore, as an initial study, our participant screening process included any veteran that completed the IOP to reach a broad understanding of device usage in our study context. Future studies could use a purposive sampling method to select veterans with specific use patterns, which could provide a deeper understanding of what drives those specific patterns and ensure that veterans with disparate use patterns were included in the data collection. Additionally, we only interviewed veterans at a single time point—after they finished the IOP program. Motivations to use the Fitbit and use of the Fitbit itself may change over time. Depending on what is tracked, the motivation to start tracking and maintain tracking may be different. It is also possible that motivations might be tied to different stages on a veteran’s journey or the stage of PTSD treatment. Future studies could investigate how wearables are used at different stages of treatment and post treatment.

Although our data did not show that gender impacted interview responses, given that the majority of our participants were male (11/13, 85% male; 2/13, 15% female), our study may not fully reflect the experience of female veterans in the program. Given that women comprised 9.4% of the total veteran population in the United States in 2015, and this is projected to steadily increase over the next 30 years, research on female veterans may become increasingly relevant [86]. Future work on the use of wearables by specifically women veterans may reveal whether there are gender-based influences on motivations for use.

We interviewed patients for their motivations and uses of the Fitbit, but clinicians may have a different set of motivations and uses for the Fitbit data. Patients generate data through wearables, but providers determine what to integrate and how to integrate into their care practice. A future study on the provider’s perspective on PGD may answer these questions and highlight opportunities and obstacles to the use of PGD in treatment. Future research could also investigate how PGD can improve therapeutic relationships and facilitate new exchanges between patient and provider.

Finally, in this study, we focused on data from the Fitbit device, as well as its mobile app, but clinical survey measures or worksheets may also be considered a form of self-logging data. Some definitions of PGD dictate that to be considered PGD, tracking must be patient-initiated as opposed to clinician-initiated, whereas others refrain from making this distinction. As the definition of PGD is only starting to be applied in the field of mental health, questions arise such as whether symptom tracking through surveys is within the realm of PGD or if it is considered a clinical tool. Further work is needed within the field of mental health on how to define PGD.

### Conclusions

Our study identified several reasons veterans undergoing intensive treatment for PTSD decided to use or not use Fitbits provided to them by the treatment program. We found that the participants of our study were motivated to use the Fitbit to increase their self-awareness, interact with fellow veterans and HCPs, and as a means of giving back to other veterans. We also found that participants of the study stopped using the Fitbit for reasons including lack of clarity of the purpose of tracking, the inability to track behaviors of interest, and challenges in the veteran-provider relationship.

As researchers continue to investigate the effectiveness of mental health treatments that integrate wearables, it is important to develop an understanding of the users of these tools. This study is one of the first to explore the patient perspective of using wearables in mental health treatment. Engaging patients will be critical to realizing the potentials of wearables and PGD in mental health treatment. We hope our research will help in future design and implementation of sociotechnical systems that integrate wearable data with clinical data.

## Appendix

Multimedia Appendix 1 Semistructured interview protocol.

## Abbreviations

CPT: Cognitive Processing Therapy
HCP: health care provider
IOP: intensive outpatient program
PA: physical activity
PCL-5: PTSD Checklist for Diagnostic and Statistical Manual of Mental Disorders, Fifth Edition
PE: Prolonged Exposure
PGD: patient-generated data
PTSD: post-traumatic stress disorder
SMI: serious mental illness
