# Supplementary material for: Veterans’ Perspectives on Fitbit Use in Treatment for Post-Traumatic Stress Disorder: An Interview Study
Source: JMIR Ment Health. 2018 Jun 15;5(2):e10415. doi: 10.2196/10415 (PMC6026306; doi:10.2196/10415)
Supplement: Multimedia Appendix 1 [file mental_v5i2e10415_app1.pdf]

Participant ID: __________
Date: _________________
Interviewer: ____________

Patient Interview

Hello! My name is ____________________, and thank you very much for calling in today.

1. Consent

[Send participant the consent form they haven't already received it. Walk through the form including the following information.]

I've asked you to volunteer to take part in this interview because I'm interested in learning more about how people that have gone through the Rush Road Home Intensive Outpatient Program either did or didn't collect information about their own health. Today, I'll be asking questions about tracking aspects of health and the use of the Fitbit. While I am affiliated with Northwestern University and not Road Home, I will be sharing my findings with Road Home which may potentially inform future processes there.

This call should take around 60 minutes. Your participation is completely voluntary. This means you don't have to participate if you don't want to and if you do agree to participate, you have the right to only answer the questions you choose to.

Just so you know, this is not a test of your skills, abilities, or knowledge – I just want to understand how someone that has gone through the program such as yourself views health tracking practices, so please give me your honest opinions. I would greatly appreciate your help. As mentioned in the consent form, I will be recording audio of your response so that I can review it later if I miss anything in my notes – the audio will be transcribed verbatim by a member of the research team or a transcription company. Potentially identifiable information will be removed to ensure confidentiality. Additionally, there may be times where I ask you questions that you may already have answered, for sake of clarity and having your responses in your own words, I may ask you to repeat yourself – please pardon me when that happens.

The potential risks of this research is minimal and confidentiality of private health information that you share with us will be maintained to the highest level and will be accessible only by me and my research team. If for any reason you are uncomfortable, you may end this session at any time. If you have any questions or concerns, you can reach the Principle Investigator at Rush University Medical Center, Dr. Alyson Zalta, at 312-563-2828 or the Principle Investigator at Northwestern University, Dr. Stephen Schueller, at 312-503-1232.

[Ask for questions and consent as documented in the consent form]

Do you have any questions before we begin?
[Answer any questions]

Ready to start recording?

[Start recording and continue]

2. Background

1. When were you at Rush for the Intensive Outpatient Program?

Let's think back to before you started the Intensive Outpatient Program at Rush:

1. Did you track any aspect of your health or well-being? This includes using pen or paper or with an app or device (i.e. weight, calorie counting, marking down number of days you ran)?
   a. [If yes] What did you track and how?
      i. Why did you start doing it?
      ii. How did you use the information?
      iii. Did you share your self-tracked data with anyone?
         1. Who? Why/why not?
   b. [If no] Why do you think you've never done any health-related tracking?
2. Let's call this type of personal health or well-being tracking "self-tracking" – For yourself, what do you see as the value of self-tracking – if there is any value at all?

3. PGD at IOP

These next few questions are about your time during the IOP.

1. Did you do any type of non-Fitbit self-tracking during your time there?
   a. [If yes] What did you track and how?
      i. How did you use the information?
      ii. Did you share it with anyone?
         1. [If yes] Who? Why?
         2. [If no] Why not?
2. Moving on to the Fitbits, how did you use it?

   [Probe on the following]:
   a. Review data
      i. Which data did you review? (For each) Why?
      ii. Was the data that you received useful to you or not?
      iii. What could have made the data more useful to you?
      iv. How often did you review this data?

v. What prompted you to review the data when you did?

vi. Do you believe reviewing your data impacted your health outcomes in any way – good or bad?

b. Share data

i. Who did you share the data with?

ii. Why did you share it?

1. What did you hope would come out of sharing the data?

2. Did you achieve what you had hoped?

iii. Through what means did you share the data? (email? write down? show in person?)

iv. When do you share the data?

v. Do you believe that sharing your data impacted your health outcomes in any way – good or bad?

c. [*If they haven't done either of the above, for <u>each</u>*]

i. Why not?

ii. If you did have to share the data with someone, who would you choose?

1. What data would you share?

2. Why would you share that data with that person?

3. How do you think they would react?

4. What do you think they would do with the data?

d. On the days you didn't wear the Fitbit, why?

3. What did you like about having the Fitbit?

4. What didn't you like about having the Fitbit?

5. Overall, how has having this Fitbit impacted you? (*positively? negatively? mentally/emotionally/physically/socially?)*

6. Why do you think you were provided a Fitbit as a part of the IOP?

7. How do you think the Road Home Program is using this data?

8. What benefits could you get out of sharing the data with Road Home?

a. How could you initiate getting that benefit?

i. Who would you initiate with?

ii. What would you do?

iii. What do you think they would do next?
iv. When would be an appropriate time to initiate?

b. How could Road Home initiate getting this benefit to you?

i. Who do you envision would be the best person to do this?

ii. What would they do?

iii. How would you react?

iv. When would be an appropriate time to do this?

9. Would you have any concerns about Road Home using your Fitbit data as part of your treatment?

a. How could this be addressed?

10. Are there other benefits you believe you could get from the Fitbit but just haven't for one reason or another?

a. [If yes] What are the barriers or challenges that prevent you from using the Fitbit for these additional benefits?

11. While at Road Home, did you have any conversations where having any self-tracking data would have been helpful (Remember, this is paper or digital tracking of any aspect of your health and well-being)? If so, what happened?

12. Did you have any discussions using self-tracked data but realized the data was not so helpful? If so, what happened?

13. Do you have any final thoughts or comments on the use or sharing of Fitbit data?

4. If time allows

14. Have you done any self-tracking since you left the program or do you plan on doing it in the future?

a. [If yes] What did you track and how?

i. How did you use the information?

ii. Did you share it with anyone?

1. Who? Why?

b. [If no] Why did you stop wearing the Fitbit?

5. Closing

Thank you so much for taking the time out of your schedule to talk to me today. Your perspective is incredibly helpful as I analyze how self-tracked data can improve the patient care process. If you have any questions or concerns, feel free to contact either of the principal investigators of this study listed on your consent form.
